# Raman spectroscopy of 2D MoS_2_ on Ti_3_C_2_ MXene: the substrate effect†

**DOI:** 10.1039/d5na00096c

**Published:** 2025-04-10

**Authors:** Ethan Pollack, Qiaohui Zhou, Elham Loni, Kenneth Agbakansi, Ahmad Majed, Fei Wang, Ali Soleymani, Melena Busse, Michael Naguib, Xin Lu

**Affiliations:** a Department of Physics and Engineering Physics, Tulane University New Orleans Louisiana 70118 USA xlu5@tulane.edu

## Abstract

We use Raman spectroscopy to study the substrate effect of Ti_3_C_2_ MXene on the lattice vibrational modes of MoS_2_. We observed redshifts in the fingerprint peaks of MoS_2_, and explained the shifts based on their vibrational nature. The shift in the in-plane E^1^_2g_ mode is attributed to the strain effect, and the softened A_1g_ mode associated with the out-of-plane vibration is caused by electron doping. In addition to monolayer MoS_2_, we show that the modulation from Ti_3_C_2_ MXene is also present in few-layer and bulk MoS_2_. Furthermore, we demonstrate that the laser-induced shift occurs even at low excitation power. Our results indicate that a detailed power-dependent measurement is indispensable for extracting the pure substrate effect from Raman spectroscopy.

## Abstract 2

Red-shifted and broadened Raman peaks of MoS_2_ reveal the strain and doping effects from Ti_3_C_2_ MXene as a substrate.

## Graphical abstract

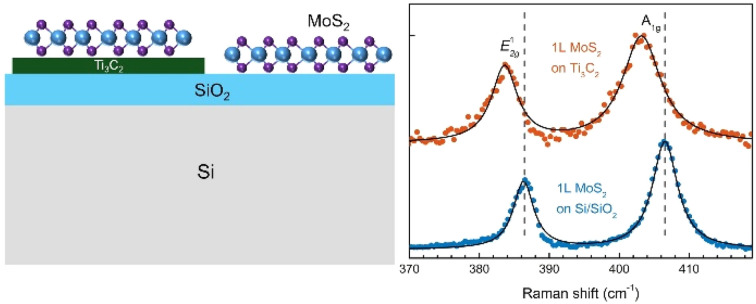

## Introduction

MXenes refer to a group of two-dimensional (2D) transition metal carbides, carbonitrides, and nitrides. With the chemical formula of M_*n*+1_X_*n*_T_*x*_ (M is a transition metal; X is carbon/nitrogen/oxygen; T denotes surface terminations), the family of MXene includes a variety of compositions and diverse properties,^1^ and hence holds great promise in energy storage,^2^ catalysis,^3^ electronics,^4^ and sensing.^5,6^ In recent years, the stacking of MXenes with other 2D materials, such as semiconducting transition metal dichalcogenides (TMDs), has attracted growing interest in nanoelectronics^7^ and catalysis. For instance, spray-coated Ti_3_C_2_ MXene films can work as metal contacts and be integrated into MoS_2_ transistor circuits for large-scale 2D electronics.^8^ Due to the larger interlayer spacing, vertically aligned MoS_2_ on Ti_3_C_2_ has been shown to be an improved catalyst in the hydrogen evolution reaction.^9^ Similarly, in tribology, the hybrid Ti_3_C_2_–MoS_2_ coatings result in super-lubricious behavior and demonstrate even better lubricity performance than MoS_2_.^10^ Despite the various applications of such hybrids, a detailed study on the interfacial interaction between Ti_3_C_2_ MXene and MoS_2_ is still lacking. In particular, how the presence of the Ti_3_C_2_ MXene film affects the lattice vibrations and structure of MoS_2_ remains largely unexplored.

**Fig. 1:**
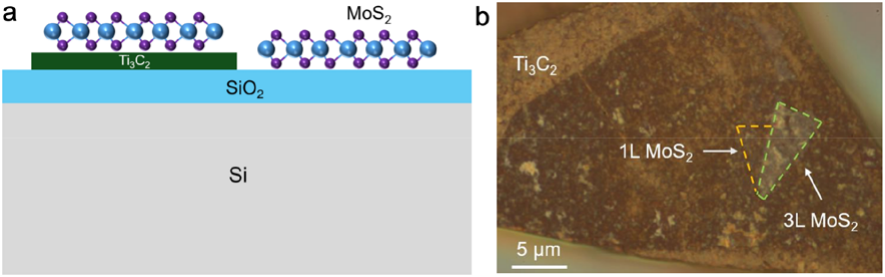
Fabrication of the MoS_2_–Ti_3_C_2_ MXene stack. (a) Schematic illustration of 1L MoS_2_ on the Ti_3_C_2_ MXene flake and the Si/SiO_2_ substrate. (b) An optical image of the MoS_2_–Ti_3_C_2_ MXene heterostructure.

With the intensive research on TMDs in the past decade, influence of the environment, including the substrate effect and the modulation from the adjacent material, has been extensively studied *via* optical spectroscopy.^11–16^ In this work, we use Raman spectroscopy as a nondestructive and rapid tool to probe the interfacial interaction between Ti_3_C_2_ MXene and MoS_2_. The Ti_3_C_2_ MXene flakes were exfoliated from Ti_3_C_2_ MXene free-standing paper (see Methods). The monolayer MoS_2_ samples were prepared by mechanical exfoliation from a bulk crystal (2D semiconductor). We fabricated the MoS_2_–Ti_3_C_2_ stack using the polydimethylsiloxane-based dry-transfer method^17^ or the pick-up technique,^18^ followed by thermal annealing. Samples prepared using the pick-up technique have a hexagonal boron nitride (h-BN) layer on top for additional protection, but we note that the modulation from Ti_3_C_2_ MXene does not depend on the top h-BN layer. Fig. 1a shows a schematic diagram of the as-stacked structure with a monolayer (1L) MoS_2_ flake placed on the Ti_3_C_2_ MXene multilayer. Another 1L MoS_2_ flake is placed on the Si/SiO_2_ substrate as a reference. In order to simulate the structures used in electronic and catalytic applications, the Ti_3_C_2_ MXene flakes we prepared are thick (Fig. 1b). The 1L MoS_2_ (∼0.7 nm) appears green/blue on the 285 nm SiO_2_ oxide layer (not shown in Fig. 1b), consistent with previous studies,^19,20^ but it “disappears” on the Ti_3_C_2_ MXene multilayer due to the optical interference effect.^11^

## Results and discussion

### Thickness-dependent Raman spectra of MoS_2_

**Fig. 2:**
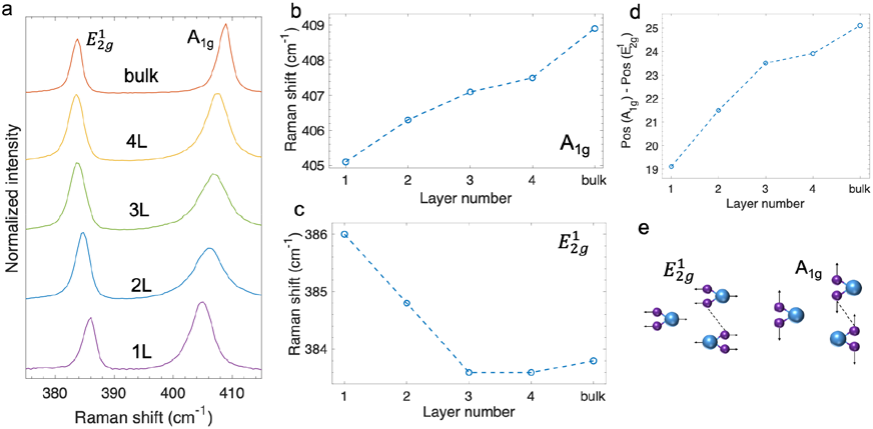
Layer-dependent Raman spectroscopy of MoS_2_ on the Si/SiO_2_ substrate. (a) Raman spectra of 1-4L and bulk MoS_2_ in the range of 375 cm^−1^ to 415 cm^−1^. (b–d) Thickness-dependent shift of the A_1g_ mode (b), E^1^_2g_ mode (c), and the energy difference of A_1g_ and E^1^_2g_ modes (d). (e) Displacement representations of A_1g_ and E^1^_2g_ modes in 1L and 2L MoS_2_.

Before discussing the interfacial interaction from Ti_3_C_2_ MXene on the lattice vibrational modes in MoS_2_, we first measured the Raman spectra of MoS_2_ on the Si/SiO_2_ substrate. Fig. 2a demonstrates the non-resonant Raman spectra of 1-4L and bulk MoS_2_ by using a 532 nm laser. We focus on the fingerprint peaks, A_1g_ and E^1^_2g_ modes. The A_1g_ peak, which involves the out-of-plane displacements of S atoms in each layer, blue-shifts from ∼405 cm^−1^ in 1L to ∼409 cm^−1^ in bulk (Fig. 2a and b). As shown in the atomic displacements (Fig. 2e), when the thickness increases, the adjacent S atoms vibrate out-of-phase. Within the classical model for coupled oscillators, the interlayer van der Waals (vdW) interaction causes larger effective forces in thicker flakes, thus stiffening the A_1g_ mode.^21,22^ The vdW model, which includes the influence of interlayer coupling on the intralayer vibration,^23^ has been introduced to explain the Davydov splitting in bulk and bilayer layered materials.^24,25^ We applied the vdW model to the A_1g_ mode in 2L MoS_2_ by following the equation *ω*_c_^2^ = *ω*_0_^2^ + Δ*ω*^2^, where *ω*_0_ is the frequency of the two uncoupled oscillators when the two neighboring sulfur atoms vibrate in-phase (A_2U_, Raman-inactive), *ω*_c_ is the frequency when neighboring sulfur atoms vibrate out-of-phase (A_1g_, Fig. 2e right) and Δ*ω* is the coupling frequency between the two entities. In the first-order approximation, Δ*ω* can be considered the energy of the interlayer breathing (LB) mode, whose energy is 40.0 cm^−1^ in our 2L MoS_2_ (Fig. S1†). With *ω*_c_ = 406.3 cm^−1^ and Δ*ω* = 40.0 cm^−1^ in 2L, we obtain *ω*_0_ = 404.3 cm^−1^, which indicates that the Raman-inactive A_2u_ mode is red-shifted compared to the A_1g_ mode in 1L (with symmetry of 
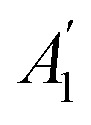
 in odd layer numbers). This result is intuitive with the classical model since the equivalent force constant from two springs in series would be smaller than either one of the oscillators.

While the thickness effect explains the shift of the A_1g_ mode, due to its in-plane vibrational nature, the E^1^_2g_ mode should be less sensitive to the thickness (Fig. 2e left). As a result, one would expect a smaller blue-shift or no-shift of the E^1^_2g_ mode with increasing thickness. The anomalous redshift (Fig. 2a and c) was once thought to originate from the dielectric effect. Nevertheless, the dielectric effect was ruled out by Lin *et al.*^26^ They placed exfoliated MoS_2_ in different solvents with dielectric constants ranging from 1.89 to 32.6 but observed no systematic Raman shifts. Later, Luo *et al.* attributed the red-shift of the E^1^_2g_ mode with increasing thickness to the surface effect, which refers to the larger Mo–S force constants at the surface of atomically thin MoS_2_ due to the loss of neighboring adjacent layers.^22^ The larger force constant increases the Raman shift of the E^1^_2g_ mode in thinner layers, causing the peak to blue-shift.^27–29^ The energy difference between the A_1g_ and E^1^_2g_ modes (Pos(A_1g_) − Pos(E^1^_2g_)) has been shown to correlate with the thickness.^21^ Our measured results show that the difference is ∼19 cm^−1^ in 1L MoS_2_ and reaches ∼25 cm^−1^ in bulk MoS_2_ (Fig. 2d), which is consistent with earlier results.^21,27–29^ The consistency indicates that unintentional strain^30,31^ and doping effects^32,33^ are absent in our exfoliated samples (compared to samples on Si/SiO_2_ only).

### Raman and PL spectra of the 1L MoS_2_–Ti_3_C_2_ MXene stack

**Fig. 3:**
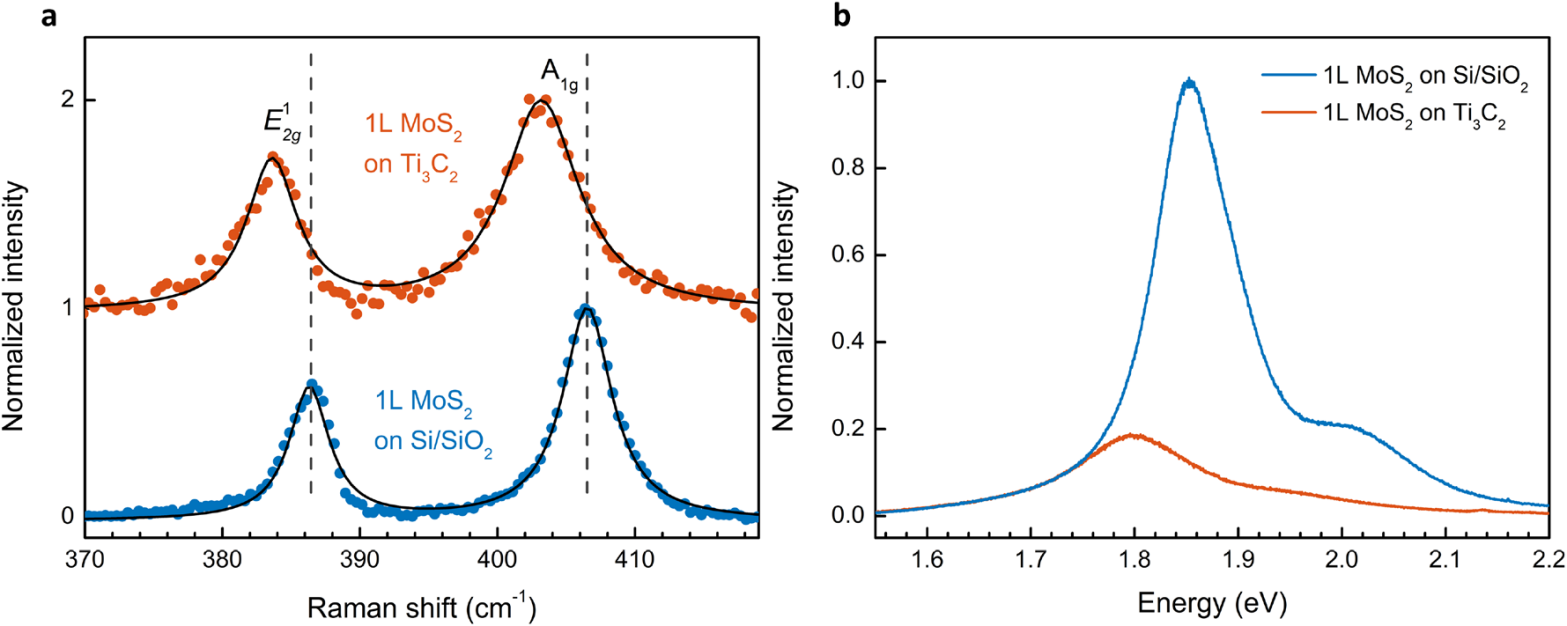
Comparison of 1L MoS_2_ on Ti_3_C_2_ MXene and 1L MoS_2_ on Si/SiO_2_. (a) Raman spectra of 1L MoS_2_ on Ti_3_C_2_ MXene and 1L MoS_2_ on Si/SiO_2_. Dots represent the experimental data and lines correspond to the Lorentzian fitting. (b) Photoluminescence (PL) spectra of 1L MoS_2_ on Ti_3_C_2_ MXene in comparison with 1L MoS_2_ on Si/SiO_2_.

We plotted the Raman spectra of h-BN capped, 1L MoS_2_ on Ti_3_C_2_ MXene and on a Si/SiO_2_ substrate. As shown in Fig. 3a, the A_1g_ and E^1^_2g_ modes from 1L MoS_2_ on Si/SiO_2_ are located at 406.5 cm^−1^ and 386.5 cm^−1^, respectively, with the energy difference being 20 cm^−1^. Compared to the Raman shifts in Fig. 2d, the larger energy difference (20 cm^−1^*vs.* 19 cm^−1^) is mainly due to the blue-shifted A_1g_ mode (406.5 cm^−1^*vs.* ∼405 cm^−1^). The stiffened A_1g_ mode resembles the thickness effect in few-layer MoS_2_, where the interlayer vdW interaction increases the effective force constant. Here, the thickness effect results from interlayer interaction with the top h-BN layer which also has a hexagonal lattice. Note that both samples (on Ti_3_C_2_ MXene and on Si/SiO_2_) are capped with h-BN, so the comparison is still valid.

Compared with MoS_2_ on Si/SiO_2_, the A_1g_ and E^1^_2g_ modes of MoS_2_ on Ti_3_C_2_ MXene red-shift by 3.3 cm^−1^ and 2.9 cm^−1^, respectively. To analyze the shifts, we started by first ruling out other possible factors. Since the redshifts persist after thermal annealing, we do not think adsorption on the surface of MoS_2_ plays a major role. Meanwhile, even if the interaction between MoS_2_ and Ti_3_C_2_ MXene introduces new resonances, the energies of both phonon modes are constant across a wide range of excitation lasers.^34^ Therefore, we further ruled out the resonance effect. The charge transfers between Ti_3_C_2_ MXene and MoS_2_ could result in a doping effect in MoS_2_. However, Chakraborty *et al.* demonstrated that the doping effect has a negligible influence on the E^1^_2g_ mode,^32^ which does not match with our observed features. As a result, we believe that the possible cause of softened peaks, especially the E^1^_2g_ mode, is strain. It is hard to completely get rid of strain in layered materials particularly when there is a lattice mismatch. In addition, the sample preparation process, whether through the pick-up technique or the polydimethylsiloxane-based dry-transfer method, may introduce even larger strain into the flakes. Rather than exhibiting uniaxial strain, when the layered material is stretched or compressed in one specific direction, the strain that MoS_2_ experiences on the Ti_3_C_2_ MXene multilayer more closely resembles to the biaxial strain with no selection on the in-plane direction. Lloyd *et al.* demonstrated that the Raman peaks red-shift linearly at a rate of 1.7 cm^−1^/% for the A_1g_ mode and 5.2 cm^−1^/% for the E^1^_2g_ mode.^31^ As the E^1^_2g_ mode corresponds to an in-plane vibration and the atoms in A_1g_ mode vibrate out-of-plane (Fig. 2e), it would be more appropriate to use the E^1^_2g_ mode to determine the in-plane strain. According to the linear rates, a redshift of 3.3 cm^−1^ from the E^1^_2g_ mode indicates that the local strain is about ∼0.6%, which should simultaneously soften the A_1g_ mode by ∼1.0 cm^−1^. Nevertheless, the observed redshift in Fig. 3a is 2.9 cm^−1^ (>1.0 cm^−1^) for the A_1g_ mode. On the other hand, the red-shifted A_1g_ mode could be attributed to the electron doping effect which hardly affects the E^1^_2g_ mode because of symmetry.^32^ If the remaining shift of ∼1.9 cm^−1^ is entirely due to the doping effect, it corresponds to an electron density of 5–6 × 10^12^ cm^−2^, which would lead to an increase in the line width by a factor of 1.5.^32^ Note that the doped electrons could come from trapped charges at the interface, as discussed in earlier reports.^11,35^ We extracted the full width at half maximum and found that while the line widths of the E^1^_2g_ mode are similar, the A_1g_ peak is broadened from 4.4 cm^−1^ on Si/SiO_2_ to 6.6 cm^−1^ on Ti_3_C_2_ MXene. The broadened line width is consistent with the electron doping effect.

We further examined the modulation of photoluminescence (PL) response by Ti_3_C_2_ MXene. As expected, 1L MoS_2_ on Si/SiO_2_ exhibits A exciton and B exciton peaks at ∼1.85 eV and ∼2.02 eV, respectively. The emission from 1L MoS_2_ on Ti_3_C_2_ MXene is suppressed. Meanwhile, the A excitonic peak is red-shifted by ∼50 meV. We first ruled out the effect from Coulomb engineering since the dielectric constant of Ti_3_C_2_ MXene^36^ is on the same order as that of SiO_2_. According to Lloyd *et al.*, the excitonic peaks in MoS_2_ shift linearly at a rate of −99 ± 6 meV/%.^31^ Based on the strain determined from the E^1^_2g_ mode, we expect a redshift of 59 meV from the A exciton, which is larger than the observed value. Moreover, the electron doping effect would result in an additional redshift on top of the strain-induced shift.^37^ The inconsistency between the shifts of the E^1^_2g_ mode and the A excitonic complex indicates that one needs to be careful with the determination of strain status, which we will discuss further in the later text.

### Raman spectra of few-layer MoS_2_ on Ti_3_C_2_ MXene

**Fig. 4:**
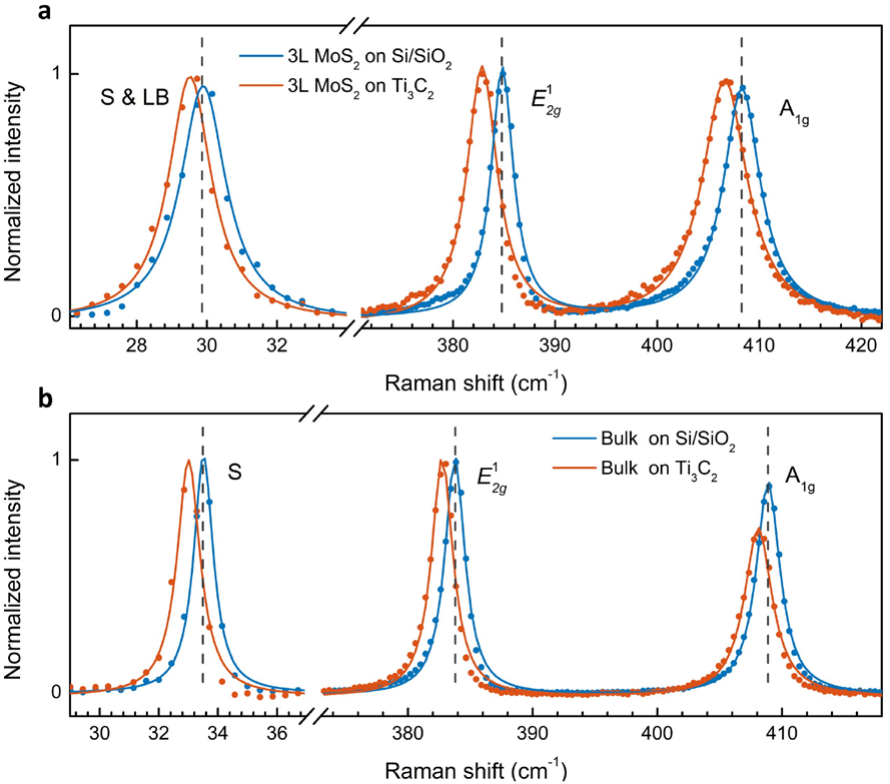
Raman spectra of 3L (a) and bulk (b) MoS_2_ on Ti_3_C_2_ MXene in comparison with the counterparts on Si/SiO_2_.

We extended our study to few-layer and bulk MoS_2_ and plotted the fingerprint peaks in Fig. 4. Similar to 1L MoS_2_, both A_1g_ and E^1^_2g_ modes soften. In addition, the low-frequency interlayer shear (S) mode also red-shifts. Compared to the intralayer in-plane mode (E^1^_2g_), a smaller shift is observed in the interlayer in-plane vibration (S mode). In 3L (bulk), the S mode red-shifts by 0.4 (0.5) cm^−1^, while the E^1^_2g_ mode is down-shifted by 2.0 (1.1) cm^−1^. Our results indicate that while in-plane strain modifies the interatomic distance and shifts the intralayer vibrational modes, it has a lower effect on the interlayer vibrations where the whole layers move as a single unit. Our observation is also consistent with an earlier report on strain-dependent low-frequency modes in MoS_2_.^38^

The A_1g_ mode in 3L and bulk also red-shifts by a slightly smaller magnitude compared to the E^1^_2g_ mode (1.7 cm^−1^*vs.* 2.0 cm^−1^ in 3L; 0.8 cm^−1^*vs.* 1.1 cm^−1^ in bulk). Note that the shift rate varies as a function of thickness. Experimentally, Hui *et al.* demonstrated that the shift ratio of E^1^_2g_ to A_1g_ is ∼1.5 in 3L under biaxial strain.^39^ If we use the E^1^_2g_ mode to determine the strain value, the strain-induced shift of the A_1g_ mode should be 1.3 cm^−1^, smaller than the observed shift of 1.7 cm^−1^. The additional redshift of 0.4 cm^−1^ could be attributed to the electron doping effect, similar to the analysis for 1L. Although the line width of the A_1g_ peak shows negligible change, different from that in 1L, the doping-induced redshift of 0.4 cm^−1^ in 3L is much smaller than that in 1L (1.9 cm^−1^). As a result, the line width is hardly affected due to the smaller doping density. The doping effect in 3L MoS_2_ is also confirmed from the PL spectra (Fig. S2†).

### Discussion on the possible laser heating effect

**Fig. 5:**
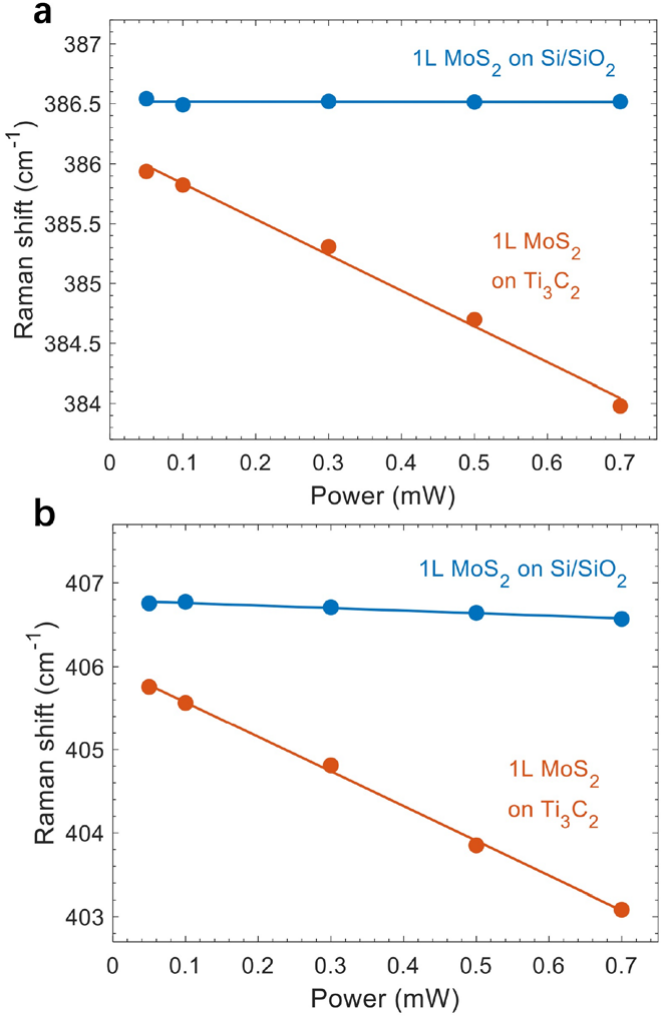
Raman peak positions as a function of excitation power. (a and b) Peak positions of the E^1^_2g_ (a) and A_1g_ (b) modes. Dots represent experimental data and lines correspond to a linear fitting.

The laser power we used for the above measurements was 0.5 mW, which is not large for MoS_2_. Nonetheless, we conducted excitation power-dependent measurements on another sample with laser power ranging from 0.05 mW to 0.7 mW. As shown in Fig. 5, the E^1^_2g_ mode exhibits a constant peak position for 1L MoS_2_ on Si/SiO_2_, while the maximum redshift from the A_1g_ peak is ∼0.2 cm^−1^, which is within the resolution of our measurements. This indicates that the laser heating effect is negligible. However, it is surprising to see that both peaks red-shift substantially as a function of power for 1L MoS_2_ on Ti_3_C_2_ MXene. Considering that the thermal conductivity of Ti_3_C_2_ MXene is higher than that of SiO_2_,^40,41^ heat dissipation should be effective *via* the Ti_3_C_2_ MXene layer. Nevertheless, the laser-induced redshifts indicate that the Ti_3_C_2_ MXene layer absorbs more heat upon laser illumination, resulting in a smaller temperature gradient and thus less effective heat transfer. We note that the power-induced shifts in both Raman peaks and PL emission are reversible (Fig. S3†), which further supports the heating effect.

As both A_1g_ and E^1^_2g_ modes soften linearly without saturation, the absence of nonlinear effects indicates that the laser-induced shifts are linear below 0.7 mW.^42^ Therefore, we used *ω*(*P*) = *ω*_0_+ *χ*_p_(*P*) to fit the power-dependent peak positions. Here, *P* is the excitation power, *ω*_0_ is the intrinsic peak position without heating effects, and *χ*_p_ is the power coefficient. For 1L MoS_2_ on Ti_3_C_2_ MXene, we found that *χ*_p_ = −3.0 cm^−1^ and *ω*_0_ = 386.1 cm^−1^ for the E^1^_2g_ mode, and *χ*_p_ = −4.2 cm^−1^ and *ω*_0_ = 406.0 cm^−1^ for the A_1g_ mode (Fig. 5, red). The extracted power coefficients are much larger than those from Si/SiO_2_-supported (Fig. 5, blue) and sapphire-supported MoS_2_,^42^ but smaller than the values observed for suspended monolayer MoS_2_.^42^ After subtracting the heat-induced shift, we still obtained a redshift of 0.4 cm^−1^ for the E^1^_2g_ mode in 1L MoS_2_, which corresponds to 0.08% strain. Such a small in-plane strain causes a negligible shift in the A_1g_ mode, which implies that the 0.8 cm^−1^ shift (corrected after subtracting the heating effect) for the A_1g_ mode is mainly due to the electron doping effect. We note that using the lowest excitation power is not necessarily the best method for measurements since destructive interference from the substrate may lower the Raman intensity and affect the signal-to-noise ratio.^11^ Instead, performing a detailed power-dependent measurement in the low and moderate range should be appropriate to subtract the laser heating effect. In addition to 1L MoS_2_, we further conducted power-dependent Raman scattering and PL measurements on 3L MoS_2_ (Fig. S4 and S5†), which also corroborate the laser heating effect. Additionally, the temperature-dependent shifts of E^1^_2g_ and A_1g_ modes from 150 K to 300 K are shown in Fig. S6,† where both peaks shift linearly, consistent with the power-dependent measurements.

## Conclusions

In summary, our work demonstrates the substrate effect of Ti_3_C_2_ MXene on MoS_2_ in Raman spectroscopy. Compared to MoS_2_ on Si/SiO_2_, we observed redshifts of the fingerprint peaks from MoS_2_ on Ti_3_C_2_ MXene and explained these shifts based on the vibrational nature of each peak. The in-plane E^1^_2g_ mode is more sensitive to strain which arises from both sample fabrication and lattice mismatch, but a small variation in strain hardly shifts the out-of-plane A_1g_ mode. We attributed the down-shifted A_1g_ mode to the electron doping effect, which is also confirmed by the suppressed and red-shifted PL emission. In addition to the monolayer, we showed that the modulation from Ti_3_C_2_ MXene is also present in few-layer and bulk MoS_2_.

Furthermore, we demonstrate that laser excitation power plays a vital role in determining the external perturbations and the magnitude of the heat-induced redshifts varies depending on the underlying substrate. As Raman spectroscopy has been widely used as a nondestructive tool to identify strain and doping effects,^43–45^ our results show that a careful power-dependent measurement is essential to subtract the laser-induced shifts and extract the pure modulation from the substrate.

Our work contributes to the understanding of interfacial interaction between Ti_3_C_2_ MXene and MoS_2_ and also provides some insights into the application of MoS_2_–Ti_3_C_2_ heterostructures. While the local heating effect from Ti_3_C_2_ MXene could potentially deteriorate the performance of MoS_2_–Ti_3_C_2_ MXene in nanoelectronics, the Ti_3_C_2_ MXene-induced strain and doping, on the other hand, may improve the performance of MoS_2_ as an electrocatalyst in the hydrogen evolution reaction.^46^

## Methods

### Synthesis of Ti_3_C_2_ MXene free-standing paper

A solution of 9 M hydrochloric acid (HCl) and 7.5 M potassium fluoride (KF) was prepared as the etching solution. 4 g of Ti_3_AlC_2_ was added slowly to 80 mL of the solution and stirred at 35 °C for 48 h using magnetic stirring in an oil bath for uniform heating. Subsequently, the solution was washed until the pH level was near neutral. The supernatant was discarded, leaving purified MXene as sediment. DI water was added to it, followed by shaking, and vacuum-filtration.

For delamination, 1 g of Ti_3_C_2_ MXene was added to 35 mL of 5 M LiCl, shaken manually for a minute, and left to soak for 24 h at room temperature. The mixture was centrifuged, and the supernatant was decanted to remove concentrated LiCl. Fresh DI water was added for washing followed by centrifugation and discarding the supernatant. The washing process was repeated three times. After washing, DI water was added, and the mixture was bath sonicated for 1 h followed by centrifugation at 5000 rpm for 1 h. The supernatant was collected and DI water was again added, followed by repeated sonication and centrifugation, until a clear supernatant was obtained. The collected supernatants were combined, shaken and vacuum-filtered to make a free-standing MXene paper. The Ti_3_C_2_ MXene multilayers were produced by mechanical exfoliation from the MXene paper. The Raman spectrum of Ti_3_C_2_ MXene paper is shown in Fig. S7,† and the Energy Dispersive Spectroscopy (EDS) mapping image of the exfoliated flakes is shown in Fig. S8.†

### Raman and PL spectroscopy measurements

We carried out the optical measurement by using a home-built setup. The measurements were conducted in a backscattering configuration, excited with a 532 nm laser. The excitation power was kept at 0.5 mW for Fig. 2–4 and varied between 0.05 mW and 0.7 mW for the laser heating effect study (Fig. 5). To reach a low-frequency Raman shift of ∼10 cm^−1^, we used volume Bragg grating filters (OptiGrate) to block the laser line. The backscattered signal was collected through a 100× objective and dispersed by an 1800 g mm^−1^ (Raman) or 300 g mm^−1^ (PL) grating before being detected by a liquid nitrogen-cooled charge coupled device (Princeton Instruments, PyLoN 1340 × 400 pixels CCD). The spectral resolution of our Raman spectroscopy measurements is ∼0.7 cm^−1^. Except for the temperature-dependent measurements, all spectra were taken at room temperature.

## Data availability

The data that support the findings of this work are available in the manuscript and its ESI.†

## Author contributions

Conceived and designed the experiments: X. L., Q. Z. and M. N. Sample fabrication: E. P., E. L., K. A., A. M., F. W., A. S. and M. B. Data acquisition: E. P., Q. Z. and K. A. Data analysis: X. L., E. P., Q. Z. and M. N. Writing – original draft: X. L., E. P. and Q. Z. Writing – review & editing: X. L., E. P., Q. Z. M.N. and E. L. All authors reviewed the manuscript.

## Conflicts of interest

There are no conflicts to declare.

## Supplementary Material

NA-007-D5NA00096C-s001
