# Supplementary material for: Raman spectroscopy of 2D MoS2 on Ti3C2 MXene: the substrate effect
Source: Nanoscale Adv. 2025 Apr 10;7(11):3456–61. doi: 10.1039/d5na00096c (PMC12016101; doi:10.1039/d5na00096c)
Supplement: NA-007-D5NA00096C-s001 [file NA-007-D5NA00096C-s001.pdf]

Supplementary Information (SI) for Nanoscale Advances.
This journal is © The Royal Society of Chemistry 2025

Supplementary information:

Raman Spectroscopy of 2D MoS_2 on Ti_3C_2 MXene: the Substrate Effect

Ethan Pollack, Qiaohui Zhou, Elham Loni, Kenneth Agbakansi, Ahmad Majed, Fei Wang, Ali Soleymani, Melena Busse, Michael Naguib and Xin Lu

Department of Physics and Engineering Physics, Tulane University, New Orleans, Louisiana 70118, United States.

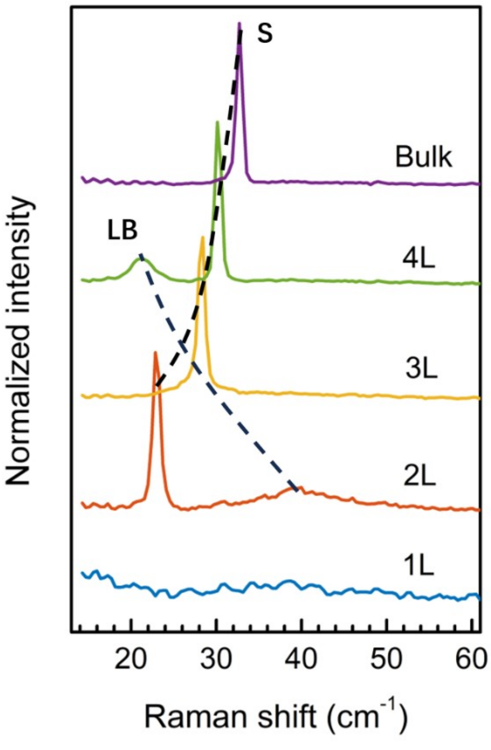

Fig. S1. Low-frequency interlayer breathing (LB) and shear (S) modes in MoS_2. Due to the nature of interlayer vibration, S and LB modes are absent in 1L. In 3L, S and LB modes are near degenerate. The LB mode is Raman-inactive in bulk MoS_2.

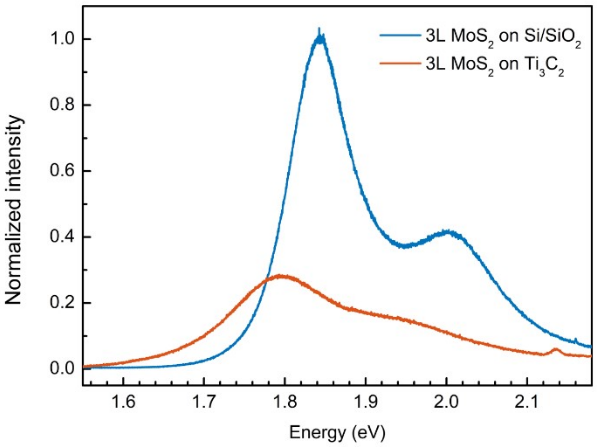

Fig. S2. Photoluminescence (PL) spectra of 3L MoS_2 on Ti_3C_2 MXene in comparison with 3L MoS_2 on Si/SiO_2.

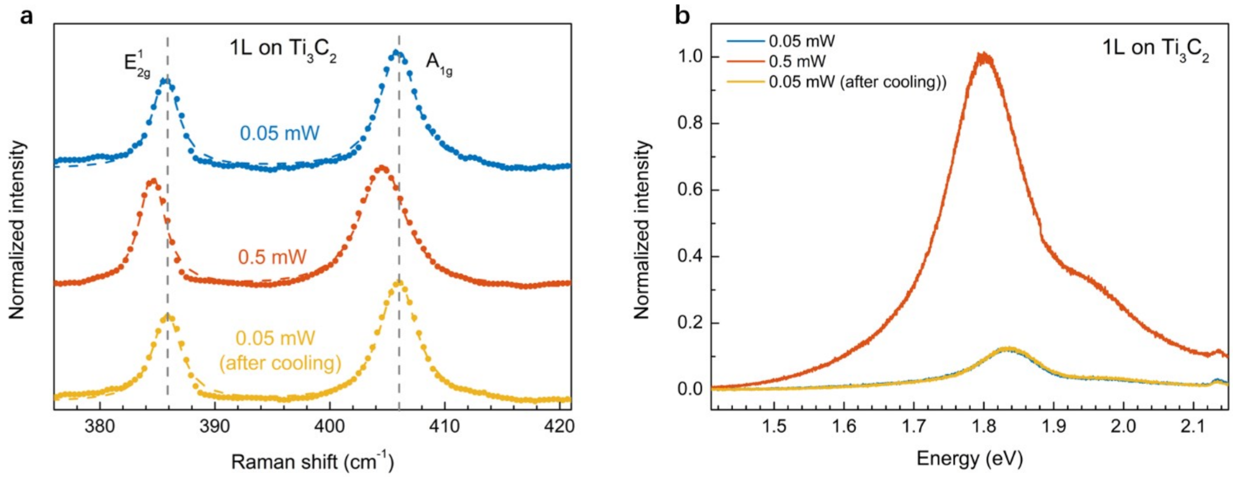

Fig. S3. Raman (a) and Photoluminescence (PL) (b) spectra of 1L MoS_2 on Ti_3C_2 MXene under excitation powers of 0.05 mW and 0.5 mW. The data from 0.05 mW (after cooling) were taken after measuring at 0.5 mW, with the laser blocked for 1 h to allow the sample to cool down.

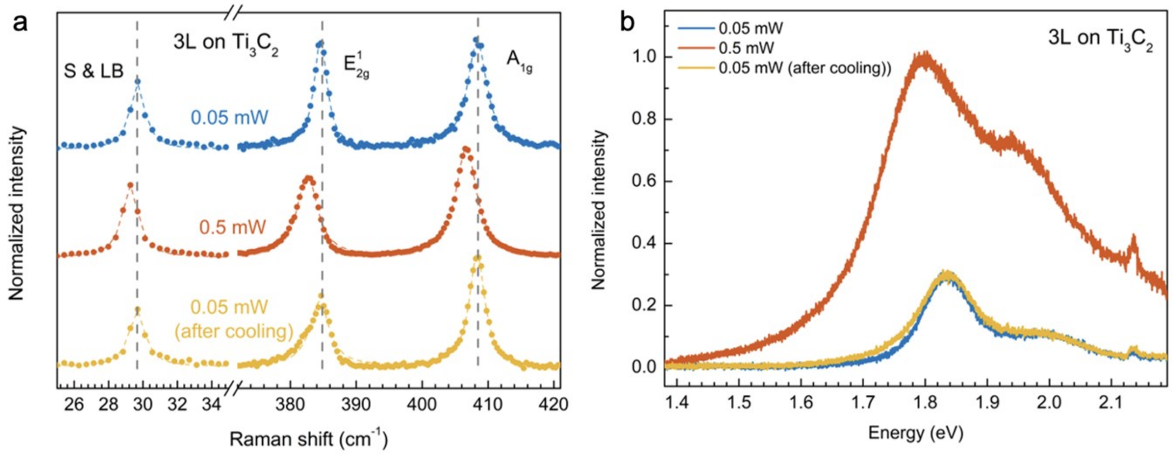

Fig. S4. Raman (a) and Photoluminescence (PL) (b) spectra of 3L MoS_2 on Ti_3C_2 MXene under excitation powers of 0.05 mW and 0.5 mW. The data from 0.05 mW (after cooling) were taken after measuring at 0.5 mW, with the laser blocked for 1 h to allow the sample to cool down.

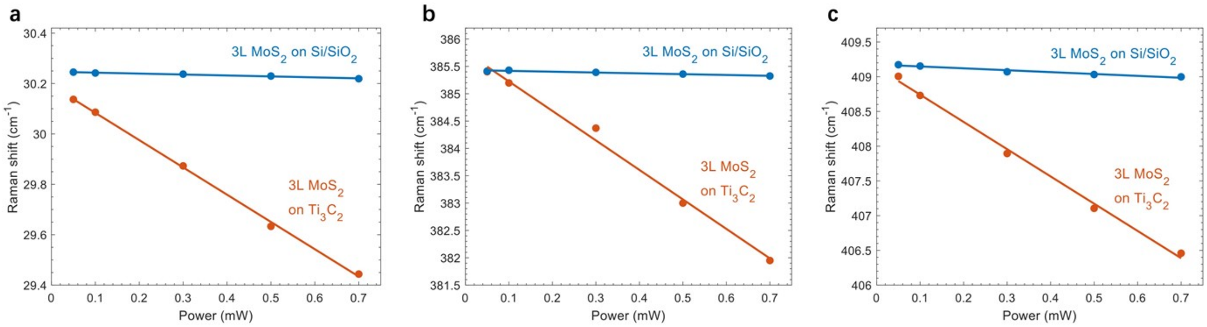

Fig. S5. Raman peak positions as a function of excitation power. (a-c) Peak positions of the S (a), E^1_{2g} (b) and A_{1g} (c) modes. Dots are experimental data and lines are from linear fits.

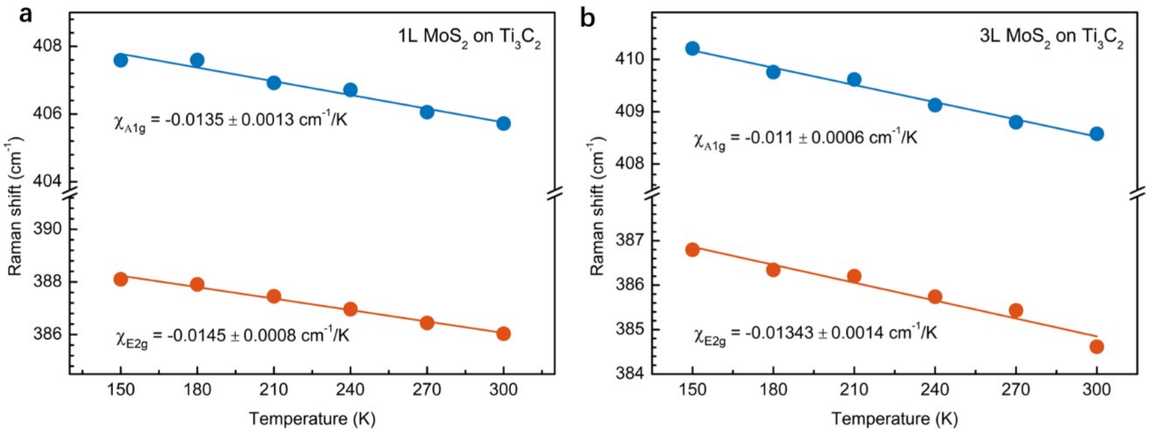

Fig. S6. Temperature-dependent Raman peak positions of 1L and 3L MoS_2 on Ti_3C_2 MXene.

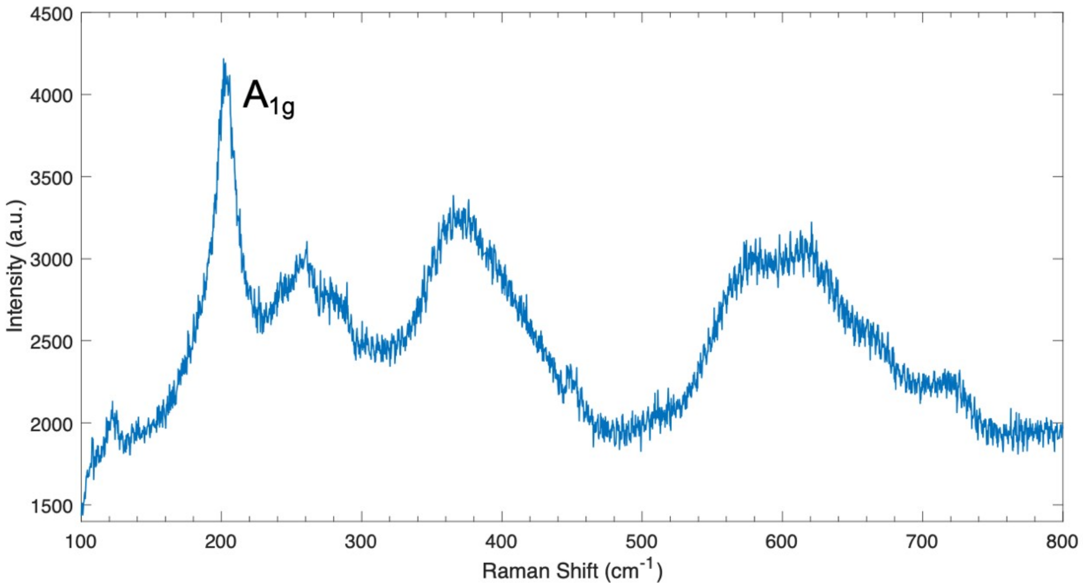

Fig. S7. Raman spectra of Ti_3C_2 MXene paper. Excitation wavelength: 633 nm.

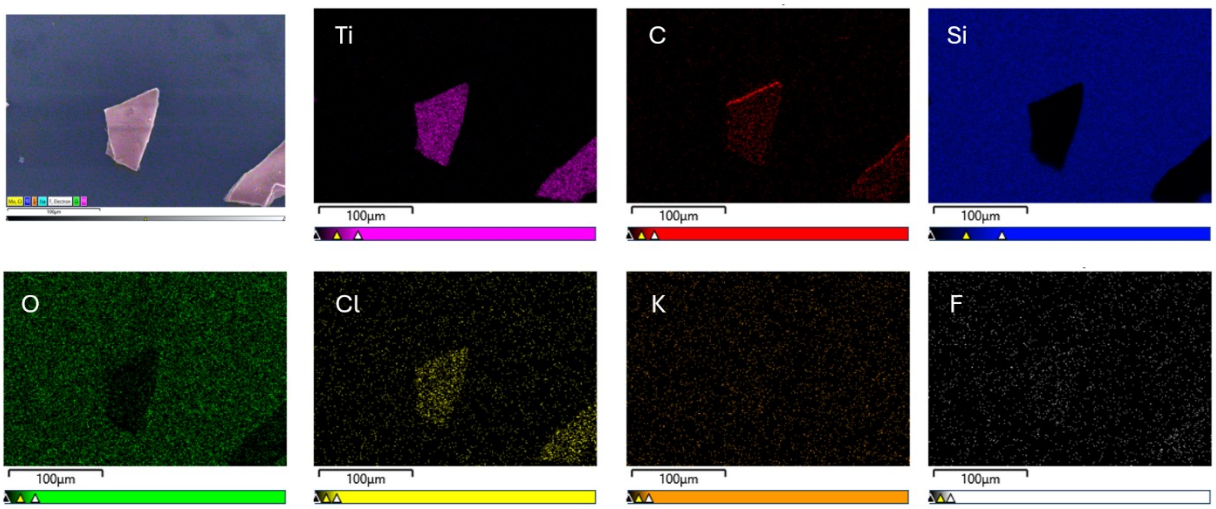

Fig. S8. Energy Dispersive Spectroscopy (EDS) mapping image of the exfoliated Ti_3C_2 multilayers on the Si/SiO_2 substrate.
